# Improving Helicobacter pylori Treatment Standards: A Quality Improvement Project Against Maastricht VI Guidelines

**DOI:** 10.7759/cureus.100058

**Published:** 2025-12-25

**Authors:** Siddharth Varier, Mark Mansingh, Disha Patel, Bhavi Desai, Yash M Shah, Mohamed A Omar

**Affiliations:** 1 Medicine, Gujarat Medical Education and Research Society (GMERS) Medical College, Vadodara, IND; 2 Medicine, Sheffield Teaching Hospital, Sheffield, GBR; 3 Medicine, Baroda Medical College, Vadodara, IND; 4 Faculty of Health Sciences, University of Nairobi, Nairobi, KEN

**Keywords:** clinical audit, eradication rates, h. pylori, quadruple therapy, quality of life improvement, triple therapy

## Abstract

Helicobacter pylori is a common infection associated with dyspeptic symptoms and long-term gastrointestinal complications when left untreated. This quality improvement project (QIP) was conducted over a six-month period with the aim of evaluating current diagnostic practice, treatment regimens, follow-up completion, and eradication outcomes in patients managed for H. pylori infection. A total of 200 adult patients were included, with a median age of 39 years and a slightly higher representation of men. Epigastric pain and burning were the most frequently reported symptoms, followed by nausea and bloating. Diagnosis was confirmed in all patients using the urea breath test or endoscopy-based methods, demonstrating appropriate compliance with recommended diagnostic standards. Bismuth-based quadruple therapy was the most commonly prescribed regimen, while clarithromycin-based triple therapy was used in a smaller proportion of cases. Follow-up attendance was recorded in 125 patients, and 90 of these underwent re-testing one month post-therapy. Successful eradication was documented in 72 of the retested patients, reflecting an 80% eradication rate in those who returned for confirmation. Among the 90 patients who underwent re-testing after follow-up, 80% (72/90) achieved successful eradication. Although the cure rate among retested patients was satisfactory, overall eradication confirmation represented only 36% of the total treated population due to incomplete follow-up and low re-testing rates. Strengthening patient education regarding the importance of treatment adherence, test-of-cure verification, and timely review may improve future outcomes.

## Introduction

Helicobacter pylori is a common, Gram-negative, spiral-shaped bacterium with multiple unipolar flagella; it is motile and produces urease. “H. pylori affects over 50% of the global population, with nearly 80% of adults experiencing infection at some point in their lives. The prevalence varies significantly across regions, while some developed countries report infection rates of <20%, developing nations such as those in Africa show prevalence exceeding 80% [1,2]. Clinically, H. pylori may present with epigastric pain or burning, nausea, bloating, belching, and loss of appetite. It is strongly associated with dyspepsia, peptic ulcer disease, mucosa-associated lymphoid tissue (MALT) lymphoma, and gastric adenocarcinoma [3].

Multiple diagnostic modalities exist for the detection of H. pylori infection [4]: (i) Invasive methods: based on endoscopic gastric biopsy specimens processed for rapid urease testing (RUT), staining, culture, histology, and molecular diagnostic techniques; (ii) Non-invasive methods: urea breath test (UBT), serology, and stool antigen test.

The Maastricht VI/Florence Consensus Report [5] provides the most comprehensive and updated recommendations for H. pylori management, highlighting preference for 14-day regimens, avoidance of clarithromycin-based triple therapy in high-resistance regions, and selection of treatment based on susceptibility patterns and patient factors [6]. Adhering to these guidelines improves eradication success and reduces long-term complications, including gastric cancer. Successful eradication has been associated with reversal of histological changes, mucosal healing, and a reduction in inflammatory mediators such as IL-1β and TNF-α [7].

Eradication outcomes depend heavily on regimen selection, treatment duration, adverse-effect burden, and, most importantly, patient counselling and compliance. Improved education regarding adherence and its long-term benefits has been shown to enhance cure rates [8,9]. Patient-centered considerations, including tolerability, affordability, and dosing convenience, further influence compliance. Masoodi et al. demonstrated higher adherence and fewer adverse effects with a two-week quadruple regimen [10], while adjunct probiotic supplementation has shown benefit in improving eradication rates [11]. Taking into account individual factors such as comorbidities, age, and prior antibiotic exposure may optimize treatment response and patient satisfaction [12].

In recent years, increasing antimicrobial resistance, particularly to clarithromycin, metronidazole, and levofloxacin, has reinforced the need for strict adherence to evidence-based regimens [13,14]. Despite well-established guidelines, variability in diagnostic choice, prescribing practices, and follow-up documentation is still common. Strengthening compliance with guideline-recommended therapy remains vital to improve eradication success, minimize antibiotic resistance, and enhance patient outcomes.

Aim of the quality improvement project

The aim of this quality improvement project (QIP) is to evaluate adherence to internationally accepted, evidence-based standards for the diagnosis and management of H. pylori infection, as recommended by the Maastricht VI/Florence guidelines. This project assesses diagnostic modality use, treatment regimen selection, follow-up review, re-testing after treatment, and final eradication outcomes. By analyzing current clinical practice and comparing it against guideline standards, the QIP intends to identify gaps in care and opportunities for intervention-driven improvement.

Through systematic review of prescribing patterns, patient documentation, counselling quality, and confirmation-of-cure rates, this project seeks to highlight practice deviations, enhance compliance, and optimize patient outcomes. Achieving higher follow-up and eradication rates is expected to reduce disease burden, minimize resistance development, and improve the cost-effectiveness of care delivery. Ultimately, the goal of this QIP is to support continuous improvement in clinical practice, strengthen adherence to guidelines, and deliver safer, more effective management for patients with H. pylori infection.

## Materials and methods

This study was a retrospective clinical QIP conducted in the Gastroenterology Outpatient Department to assess compliance with H. pylori diagnosis, treatment and eradication confirmation in accordance with the Maastricht VI/Florence Consensus Report guidelines. The QIP covered a six-month period from 1 June 2024 to 1 December 2024.

Study population

A total of 200 adult patients diagnosed with H. pylori infection and initiated on eradication therapy were included. All eligible patients who met the inclusion criteria during the audit period were included using consecutive sampling. No selective or convenience sampling was performed.

Inclusion Criteria

Patients were eligible if they: (i) Adults aged >18 years of age; (ii) Presented with upper gastrointestinal symptoms including epigastric pain, burning, bloating, nausea, belching or loss of appetite; (iii) Had H. pylori infection confirmed by one diagnostic modality: UBT, endoscopic biopsy with RUT, or histopathology.

Exclusion Criteria

Exclusion criteria were (i) Age <18 years (due to pediatric management guidelines differ significantly from adult protocols); (ii) Inpatients; (iii) Complicated duodenal ulcer (bleeding, perforation); (iv) Severe systemic disease (cirrhosis, malignancy etc.); (v) Use of proton pump inhibitors (PPIs)/H2 blockers within four weeks of endoscopy because these medications can suppress H. pylori density and reduce diagnostic test sensitivity particularly for RUT, UBT, and biopsy-based tests.

Data source & variables

Patient data were retrieved using Electronic Medical Records (EMRs) and ICD-10 diagnostic codes associated with H. pylori presentation including R10.13 (Epigastric Pain), R12 (Heartburn), R11.0 (Nausea), R10.9 (Unspecified Abdominal Pain), R10.10 (Upper Abdominal Pain), R63.0 (Loss of Appetite) and R14.0 (Abdominal Distension). ICD-10 coding was used as an initial screening tool and all identified cases were subsequently validated through manual chart review to ensure accuracy and minimize misclassification.

Extracted Parameters 

Excluded parameters* were *(i) Demographics: age and gender; (ii) Clinical presentation; (iii) Diagnostic method used; (iv) Therapeutic regimen prescribed (Quadruple vs Triple therapy); (v) Follow-up status and re-testing after treatment; (vi) Eradication confirmation outcome

QIP criteria & standards

Table 1 summarizes the predefined QIP criteria and corresponding target standards, which were derived from the Maastricht VI/Florence Consensus Report and used to benchmark departmental performance.

**Table 1:** Compliance Measured against Maastricht VI/Florence Guideline Quality Standards All QIP standards were derived from the Maastricht VI/Florence guidelines, including target rates for diagnostic confirmation, retesting timing, and expected eradication threshold QIP: Quality improvement project

QIP Standard	Target Compliance
Confirmed diagnosis before treatment	100%
Follow-up ≥30 days after treatment	90%
Re-testing ≥1-month post-therapy	85%
Successful eradication after first-line therapy	85%

Data handling and analysis and patient counseling**

Data were entered into Microsoft Excel and analyzed using descriptive statistics. Categorical variables were summarized using frequencies and percentages. Continuous variables were reviewed for distribution and reported as medians with ranges, as the dataset was not normally distributed. No inferential statistics or confidence intervals were calculated, as the QIP aimed to assess adherence to standards rather than test hypotheses, hence areas of non-compliance were evaluated to guide opportunities for practice improvement. This QIP followed ethical conduct. Patient identity was anonymized and confidentiality maintained. Ethical approval was taken and approved by the institutional human ethics committee. Patients received verbal instructions regarding medication adherence, potential side effects, and the importance of follow-up testing one month after treatment.

## Results

Two hundred clinically confirmed patients with H. pylori infection were chosen, 109 male patients and 91 female patients, after careful consideration of the pre-defined inclusion and exclusion criteria between June to December 2024. The total of 200 patients included in the QIP represented a broad adult population, with a median age of 39 years (range 18-60 years). Overall, 54.5 % (n=109) of patients were male and 45.5 % (n=91) were female. No patients outside this age range were identified and there were no exclusions based on demographic characteristics.

**Figure 1:**
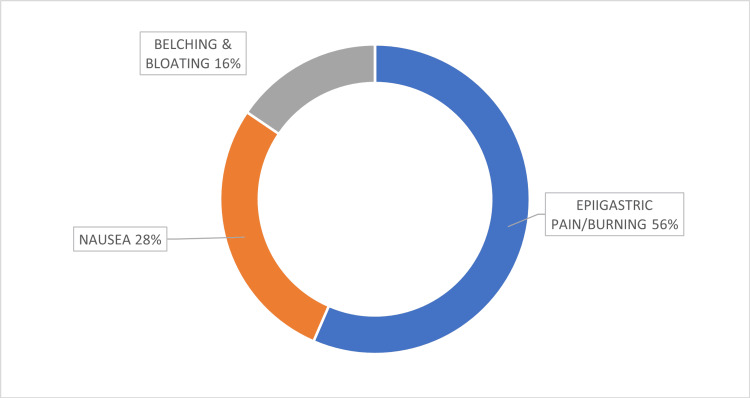
Presenting Symptoms (n=200)

The most common indications for testing were the presence of dyspeptic symptoms like epigastric pain/burning, nausea, bloating and belching. Figure 1 illustrates the distribution of presenting symptoms.

The most common symptom reported was epigastric pain/burning (56%) followed by nausea (28%), belching and bloating (16%). Patients underwent diagnostic testing using UBT (118) and endoscopy (82), of which 13 histopathology examinations and 69 RUTs were performed.

**Figure 2:**
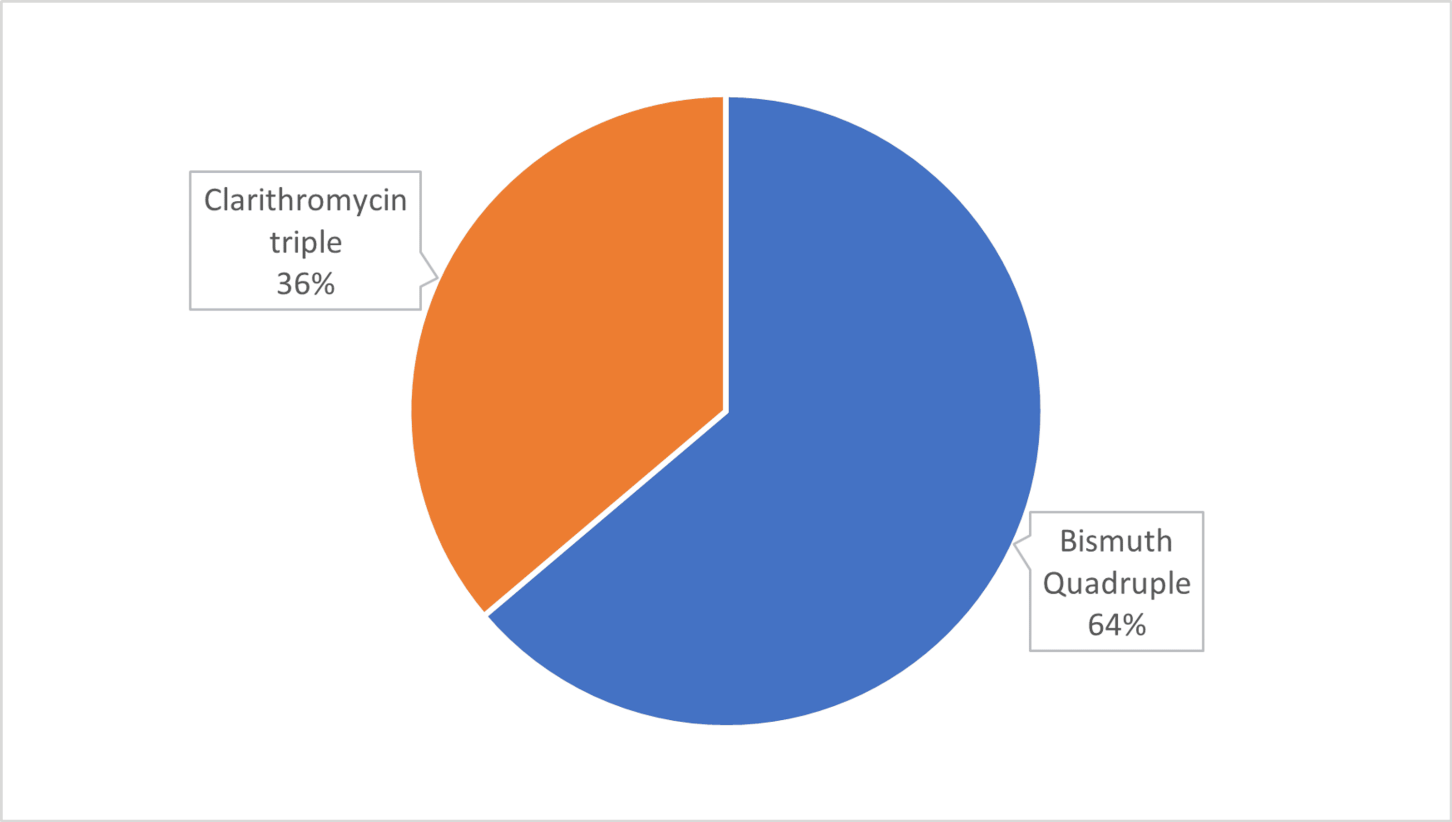
Treatment Regimen among 200 Patients

The treatment regimen was prescribed according to the Maastricht VI/Florence consensus report. The majority received the first line Bismuth quadruple therapy (124, 62 %) which included PPIs, bismuth, tetracycline and metronidazole and 76 (38%) received clarithromycin triple therapy which included PPIs, clarithromycin and amoxicillin. Figure 2 demonstrates the distribution of treatment regimen.

From the 200 patients, 125 came for the follow-up after ≥30 days of completed treatment; 45% of the total cohort (90/200) underwent re-testing after one month of the completed treatment. The remaining patients either declined re-testing, were asymptomatic and opted against further evaluation, or were lost to follow-up. Among the total study population, 36% (72/200) achieved confirmed eradication of H. pylori infection (Table 2).

**Table 2:** Summary of the Study Population (n=200) PPI: Proton pump inhibitor

Variable	Category/Description	Number (n)	Percentage (%)
Total	Patients	200	100
Gender	Male	109	54.5
	Female	91	45.5
Common presenting symptoms	Epigastric pain/burning	113	56.5
	Nausea	56	28
	Bloating / belching	31	15.5
Diagnostic methods used	Urea breath test	118	59
	Endoscopy with rapid urease test	69	34.5
	Histopathology (biopsy)	13	6.5
Treatment regimen prescribed	Bismuth quadruple (PPI + Bismuth + Tetracycline + Metronidazole)	124	62
	Clarithromycin triple (PPI + Clarithromycin + Amoxicillin)	76	38
Follow-up attendance	Returned for follow-up	125	62.5
Re-tested post-treatment	Underwent re-test (1 month after antibiotics)	90	45
Successful eradication	Confirmed H. pylori negative	72	36.0 (of total)/80% (of re-tested)

**Figure 3:**
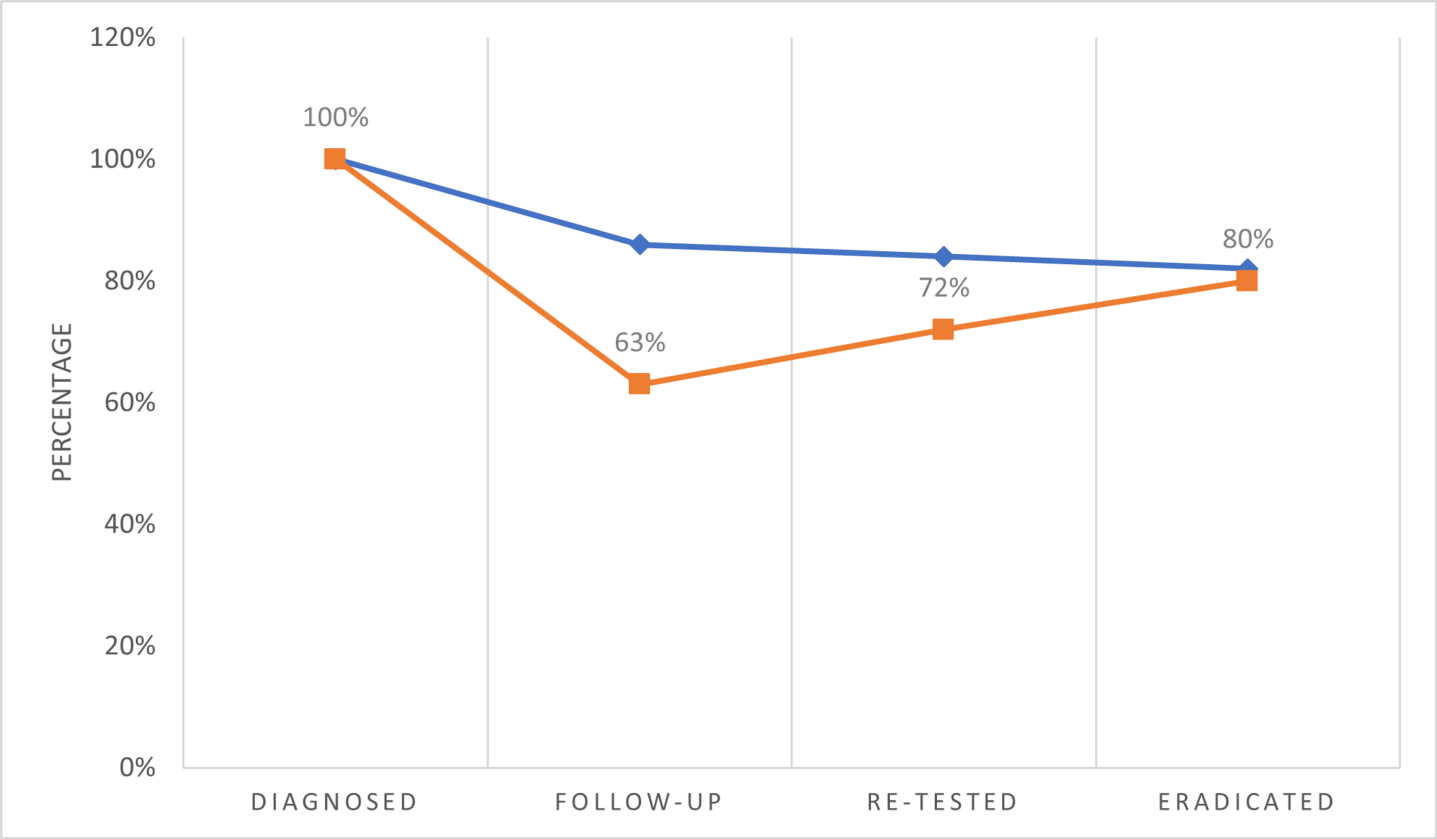
Summary of the Study Population (n=200)

The target value to reach the achievement score is established according to the Maastricht VI/Florence consensus report. Figure 3 displays follow-up and retesting rates for the cohort.

**Figure 4:**
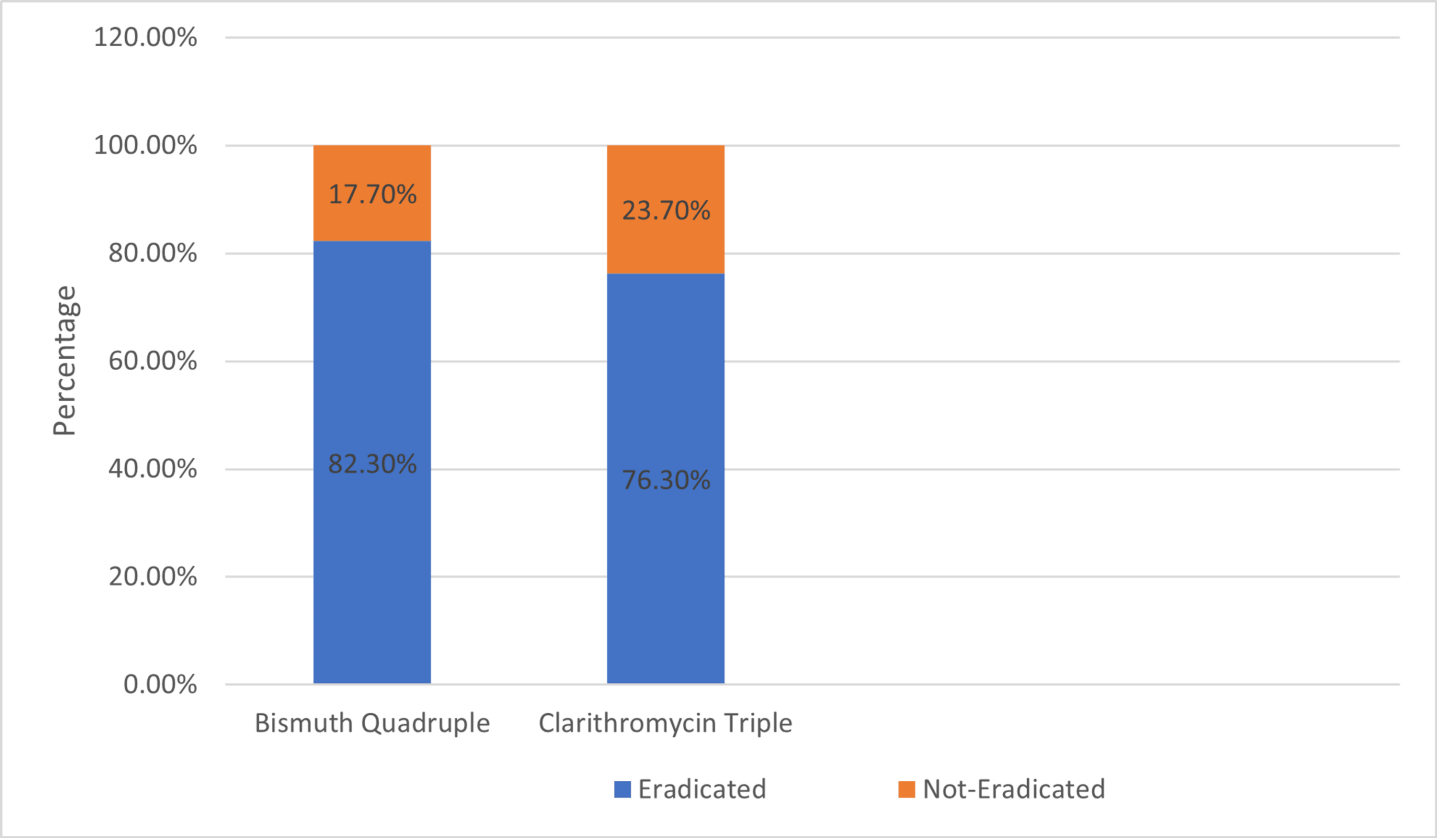
Eradicated vs Non-Eradicated by Regimen

Among 200 patients, 124 (62.0%) received bismuth quadruple therapy and 76 (38.0%) received clarithromycin triple therapy. Eradication was confirmed in 82.3% of patients treated with bismuth quadruple therapy and 76.3% of those treated with clarithromycin triple therapy. Figure 4 shows eradication rates by regimen in the form of a stacked bar-graph.

## Discussion

This QIP evaluated adherence to guideline-recommended diagnosis, treatment, and follow-up for H. pylori infection over a six-month period in a tertiary Gastroenterology outpatient setting. A total of 200 patients met the inclusion criteria, with a median age of 39 years and a slight male predominance (54.5%). Symptom distribution demonstrated a typical dyspeptic presentation with epigastric pain/burning being most frequent, followed by nausea and bloating, which is consistent with the described classical symptom profile of H. pylori-associated gastritis in published literature. The diagnostic pattern observed in this study also aligned with recommendations and favors the use of non-invasive modalities where feasible, with UBT utilized in 118 patients (59%), while the remaining cases underwent endoscopy-based RUT or biopsy [15]. All patients received a confirmed diagnosis prior to therapy initiation, achieving the QIP target for diagnostic compliance.

Treatment practice reflected strong adherence to the Maastricht VI/Florence guidance. The majority (62%) received first-line bismuth-based quadruple therapy, while the remainder were treated with clarithromycin-based triple therapy, appropriate in settings of uncertain clarithromycin resistance. The eradication outcome provides a key interpretive point: while eradication was confirmed in 80% of patients who returned for testing, the overall eradication relative to the entire treated cohort was only 36%, reflecting the major clinical gap, follow-up loss and incomplete re-testing.

International comparisons allow contextualization of our findings. Weng et al. demonstrated that successful eradication leads to reversal of inflammatory and histological mucosal injury, including reduction in IL-1β and TNF-α activity, highlighting the long-term benefit of complete cure for gastric mucosal health [7]. This shows the importance of achieving a higher full-cohort eradication rate rather than cure confirmation in only those retested. Masoodi et al. reported eradication rates of 79-81% using two-week quadruple therapy in Southern Iran [10], a figure similar to the 82.3% eradication observed among our quadruple-therapy patients. Meanwhile, a Singapore clinical audit. demonstrated a national benchmark eradication rate of approximately 92% [16], considerably higher than the 80% confirmed cure observed in our retested subset. A plausible reason for this difference may be the relatively low re-attendance and incomplete re-testing rate in our cohort, as eradication confirmation occurred in <50% of the total sample.

Comparatively, Fekadu et al. reported pooled African eradication results around 79%, but emphasized regional variation influenced by antibiotic resistance, patient adherence, co-morbid illness and prior treatment exposure [12]. The similarity between their eradication range and our re-tested cure rate suggests that our pharmacological strategy is clinically sound, but system-level inefficiencies, primarily follow-up loss, constitute the limiting factor. The Clarithromycin-triple regimen demonstrated a lower cure percentage (76.3%) than bismuth quadruple, consistent with global data describing declining clarithromycin efficacy in high-resistance regions [17]. Both Masoodi and Fekadu highlight this trend [10,12], supporting our preference for quadruple therapy when unclear resistance profiles exist.

The QIP further revealed that only 62.5% returned for follow-up, and only 45% underwent re-testing, falling substantially below target standards of 90% and 85%, respectively. Considering that eradication confirmation is essential to preventing ulcer recurrence, MALT lymphoma, and gastric cancer, this represents the most critical quality gap. Literature reinforces this. Tang et al. emphasize that success is strongly dependent on patient adherence, prior antibiotic exposure, and structured reassessment [9]. Our incomplete retesting therefore likely led to underestimation of true cure rates across the population and, more importantly, created missed opportunities to re-treat non-responders early.

Several improvement avenues arise from these findings. Our current treatment selection meets the guidelines; however, performance could increase with structured recall systems, SMS-based follow-up reminders, nurse-led re-test scheduling, and documented patient counselling, stressing the need for post-therapy confirmation. The introduction of probiotic-supplemented treatment, as suggested by Liang et al., may further reduce adverse effects and improve compliance in future cycles [11]. Protocol amendments could also include automatic scheduling of re-testing at the prescription point, integration of reminders into EMRs, and inclusion of treatment-completion counselling leaflets to improve patient engagement [18].

Limitations

This single-center, retrospective audit relied on the accuracy of EMRs, which may have limited capture of patient-reported barriers such as socioeconomic constraints, external testing, or incomplete counseling, which may have affected follow-up and eradication outcomes. Follow-up and re-testing rates were lower than recommended, further limiting assessment of treatment success. Antibiotic resistance patterns were not evaluated, restricting the interpretation of regimen effectiveness. Additionally, the retrospective design did not allow systematic assessment of patient-reported barriers, such as socioeconomic factors or adherence challenges, which may have influenced follow-up and outcomes.

## Conclusions

This six-month QIP, conducted among patients diagnosed with H. pylori infection, demonstrated variable levels of patient follow-up attendance and re-testing compliance. Although eradication among those re-tested was satisfactory, comparative review against international benchmarks highlighted a considerable gap between expected and achieved outcomes. This reinforces the need for review and optimization of the current management pathway to increase complete eradication documentation and long-term disease resolution.

Enhanced patient education and awareness stand out as critical components in improving eradication success. Emphasis should be placed on early detection, adherence to therapy, the full 14-day treatment duration, and selection of guideline-appropriate regimens tailored to patient profile and antibiotic exposure history. Strengthening patient instruction, reinforcing follow-up scheduling and improving re-testing completion may help align the center’s outcomes with international standards and contribute to improved long-term clinical control of H. pylori infection.
